# TLR7 Deficiency Reveals Compartment-Specific Respiratory–Systemic Immune Responses During Severe Influenza A Virus Infection

**DOI:** 10.3390/cells15211922

**Published:** 2026-10-22

**Authors:** Mark A. Miles, Stella Liong, John J. O’Leary, Doug A. Brooks, Stavros Selemidis

**Affiliations:** 1 Centre for Respiratory Science and Health, School of Health and Biomedical Sciences, RMIT University, Bundoora, VIC 3083, Australia; stella.liong@rmit.edu.au; 2 Discipline of Histopathology, School of Medicine, Trinity Translational Medicine Institute (TTMI), Trinity College Dublin, D08 XW7X Dublin, Ireland; olearyjj@tcd.ie (J.J.O.); douglas.brooks@adelaide.edu.au (D.A.B.); 3 Sir Patrick Dun’s Laboratory, Central Pathology Laboratory, St James’s Hospital, D08 XW7X Dublin, Ireland; 4 CERVIVA Research Consortium, Trinity College Dublin, D08 XW7X Dublin, Ireland; 5 School of Pharmacy and Biomedical Science, College of Health, Adelaide University, North Terrace, Adelaide, SA 5001, Australia

**Keywords:** Toll-like receptor 7, influenza, inflammation, infection, immunity

## Abstract

**Highlights:**

**What are the main findings?**

- TLR7 deficiency was associated with compartment-specific differences in innate and adaptive immune responses across the respiratory tract, blood and spleen during influenza A virus infection.
- Differences included altered myeloid and dendritic cell profiles, reduced systemic cytotoxic T cell activation and inflammatory markers, and changes in mucosal and systemic antibody isotype profiles.

**What are the implications of the main findings?**

- The findings highlight the interconnected nature of respiratory and systemic immune responses during severe influenza A virus infection, with differences between WT and TLR7-deficient mice extending across multiple tissue compartments.
- These findings provide a basis for investigating how TLR7 status, viral burden and compensatory immune-sensing pathways contribute to antiviral immunity and inflammation, including whether TLR7 modulation can alter inflammatory responses while preserving effective antiviral defense.

**Abstract:**

Influenza A virus (IAV) infection can progress from a localized respiratory illness to severe disease characterized by pulmonary inflammation, systemic cytokinemia, acute-phase responses and multi-organ pathology. Toll-like receptor 7 (TLR7) is a key sensor of viral single-stranded RNA and an important component of antiviral immunity; however, its role in extending immunity from the respiratory tract to peripheral compartments during severe IAV infection remains incompletely understood. Here, we investigated the relationship between TLR7 and innate, cellular and humoral immune responses across respiratory and peripheral compartments during IAV infection. Using TLR7-deficient mice infected with the highly pathogenic PR8 H1N1 strain, we profiled immune responses in nasal tissue, bronchoalveolar lavage fluid, lung, blood and spleen, together with cytokine production, C-reactive protein levels, T cell activation and antibody isotype responses. Loss of TLR7 was accompanied by compartment-specific alterations in innate immunity, including differences in pulmonary macrophage and dendritic cell responses, and altered plasmacytoid dendritic cell distribution across respiratory and peripheral compartments. Reduced virus-specific CD8+ T cell expansion, lower IFNγ and TNFα production by T cells, and diminished circulating inflammatory cytokines and C-reactive protein were also observed. In parallel, altered humoral responses included reduced mucosal IgA, systemic IgM and Th1-associated IgG2a responses, together with changes in antibody isotype distribution. Collectively, these findings demonstrate that TLR7 deficiency is associated with broad, compartment-specific alterations in innate and adaptive immune responses during established IAV infection, extending across the respiratory tract, blood and spleen and correlating with disease severity. These findings support a role for TLR7-associated signaling in shaping the broader respiratory–systemic immune response, with its presence linked to both antiviral immune engagement and the magnitude of systemic inflammation during severe IAV infection.

## 1. Introduction

Influenza A virus (IAV) infects up to one billion individuals annually, with morbidity and mortality disproportionately affecting vulnerable populations in whom infection progresses to the lower respiratory tract [1]. Although severe influenza is often viewed primarily as a respiratory infection, the clinical phenotype and severe disease can extend well beyond the lung. Patients with a severe IAV infection develop exaggerated pulmonary inflammation together with circulating cytokines, acute-phase responses, vascular dysfunction and, in the most severe cases, multi-organ involvement [2]. This indicates that local respiratory infection can evolve into a broader systemic inflammatory and pathological disorder. However, the mechanisms that determine when and how respiratory immune activation propagates into systemic compartments remain poorly defined.

The localized lung-to-systemic transition is characterized by elevated inflammatory mediators both within the respiratory tract and in the circulation. Increased concentrations of mediators such as interferon-γ (IFNγ), tumor necrosis factor (TNF), and interleukins correlate with respiratory failure, multi-organ dysfunction, and can be associated with significant mortality [3,4,5]. In parallel, acute-phase reactants such as C-reactive protein (CRP) are elevated, reflecting widespread systemic inflammation, which can lead to tissue injury [6]. These systemic inflammatory responses are consequently not merely by-products of infection but are increasingly recognized as central drivers of disease progression, contributing to vascular dysfunction, tissue damage, and impaired physiological homeostasis beyond the lung [7]. Together, these observations highlight that IAV disease progression is driven by coordinated, multi-compartment immune responses that extend from the respiratory tract into the systemic circulation. The development of severe disease is therefore shaped not only by viral replication but also by how inflammatory signals are propagated and amplified in different tissues/compartments. While early innate immune responses are essential for viral control, excessive or poorly regulated inflammatory signaling via the “cytokine storm” amplifies tissue damage and worsens disease outcomes [8]. Importantly, inflammatory responses within the lung do not always associate with equivalent systemic inflammation, suggesting that specific mechanisms govern spatial coordination and dissemination of immune response during a severe infection.

Toll-like receptor 7 (TLR7) is a key sensor of IAV-derived single-stranded RNA (ssRNA) and a potent driver of antiviral, pro-inflammatory, innate and humoral immune responses during an IAV infection [9]. Our previous work and that of others have shown that TLR7 signaling promotes inflammatory cytokine production, immune cell recruitment, antigen-presenting cell activation and Th1-biased adaptive immunity within the respiratory tract [10,11,12,13,14]. However, excessive or sustained TLR7 activation can also exacerbate virus-induced pulmonary inflammation and impair respiratory function [15,16,17,18], highlighting its dual role in antiviral defense and immunopathology. Beyond the local pulmonary effects, direct TLR7 stimulation also has the capacity to drive systemic immune activation [19], and dysregulated TLR7 signaling has been strongly implicated in systemic inflammatory and autoimmune disease [20]. These observations position TLR7 as a plausible molecular link between local respiratory viral sensing and broader systemic immune activation. However, it remains unclear how TLR7 influences the immune cascade from viral detection and antigen processing through to innate recruitment, adaptive immune activation and systemic inflammatory pathology during severe IAV infection.

We therefore investigated whether TLR7 deficiency was associated with the spatial organization and magnitude of immune responses during established severe IAV infection. Employing a TLR7-deficient mouse model infected with the highly pathogenic IAV PR8 (H1N1) strain, we performed comprehensive immune profiling across respiratory and peripheral compartments, including the nasal cavity, lungs, bronchoalveolar lavage fluid (BALF), blood, and spleen. We further assessed circulating cytokines, virus-specific T cell responses and antibody responses to determine how the absence of TLR7 is associated with innate and adaptive immunity across these compartments. This approach revealed compartment-specific alterations extending from the respiratory tract to systemic immune responses, providing insight into how TLR7-associated antiviral immunity relates to the broader inflammatory phenotype of severe IAV infection.

## 2. Materials and Methods

### 2.1. Mouse Model and Influenza Virus Infection

C57BL/6J mice (RRID:IMSR_JAX:000664) were sourced from the Animal Resources Centre (Murdoch, WA, Australia). TLR7-deficient mice (B6.129S1-Tlr7tm1Flv/J; RRID:IMSR_JAX:008380), obtained from The Jackson Laboratory (Bar Harbor, ME, USA), were maintained as a homozygous colony at the RMIT University animal research facility (Bundoora, VIC, Australia). Animals were maintained under a 12 h light/12 h dark cycle with unrestricted access to food and water.

Male mice aged 14–18 weeks were anesthetized by inhalation of isoflurane before intranasal administration of either phosphate-buffered saline (PBS) or 50 plaque-forming units (PFU) of IAV strain A/Puerto Rico/8/1934 H1N1 (PR8) in 35 µL. PBS-treated wild-type (WT) and TLR7-deficient mice were included as genotype-matched uninfected controls. The study comprised 50 mice, distributed across experimental groups of 4–8 animals, as indicated in the relevant figure legends. Body weight and clinical condition were recorded daily following inoculation. Clinical severity was graded on a 0–3 scale based on the combined assessment of weight loss, lethargy, ruffled fur and labored breathing. Animals were euthanized at 7 or 14 days post-infection (dpi) by intraperitoneal administration of ketamine (180 mg/kg) and xylazine (32 mg/kg). All procedures were approved by the RMIT University Animal Ethics Committee (approval number 23328) and conducted in accordance with the Australian National Health and Medical Research Council guidelines for animal experimentation.

### 2.2. Collection and Processing of Tissue Samples

Respiratory tissues, peripheral blood and spleens were collected for immunological analyses. Nasal tissue, comprising the nasal cavity and turbinates, and lung tissue were minced with scissors and digested in 1% Liberase (Sigma, VIC, Australia) at 37 °C with shaking at 700 rpm. Digestion times were 30 min for nasal tissue and 45 min for lung tissue. The resulting preparations were homogenized using a 21-gauge needle and passed through 40 µm cell strainers to obtain single-cell suspensions. Bronchoalveolar lavage fluid (BALF) was collected as previously described to sample the lower airways [13]. Cells were separated from the BALF by centrifugation and retained for flow cytometry, while the cell-free supernatant was snap-frozen and stored at −80 °C for subsequent biochemical analyses. Splenic cell suspensions were generated by mechanically dispersing the tissue through a 40 µm mesh filter. Blood was obtained by cardiac puncture using Clexane-loaded syringes. Red blood cells were removed from all sample preparations using ammonium-chloride-potassium lysis buffer. Following lysis, cells were washed and resuspended in fluorescence-activated cell sorting (FACS) buffer consisting of PBS supplemented with 2.5% fetal bovine serum (FBS; Scientifix, VIC, Australia) before immunophenotyping.

### 2.3. Flow Cytometric Analysis of Immune Cell Populations

Flow cytometry analysis was conducted as previously performed [18], extending that methodology to additional tissues and immune readouts. Single-cell suspensions were incubated with fluorescently labeled anti-mouse antibody cocktails prepared in FACS buffer for 30 min on ice. Unless otherwise indicated, antibodies were obtained from BioLegend (Wangara, WA, Australia). The panel comprised B220-FITC (RA3-6B2), CD11b-BV421 and CD11b-APC-Cy7 (both M1/70), CD11c-PE-Cy7 (N418; eBioscience, San Diego, CA, USA), CD4-APC-Cy7 (GK1.5), CD44-FITC (IM7; BD Biosciences, Melbourne, VIC, Australia), CD45-Alexa Fluor 700 and CD45-BV650 (both clone 30-F11), CD8a-PerCP (53-6.7), CD69-BV650 (H1.2F3), F4/80-PE (BM8; eBioscience) Ly6C-PerCP (HK1.4), Ly6G-APC-Cy7 (1A8), MHC-II-APC (M5/114.15.2), NK1.1-BV605 (PK136) and PDCA-1-Pacific Blue (217108). Influenza-specific CD8+ T cells were identified using an APC-conjugated DbPA224 peptide tetramer synthesized by the Innate Immunity and Anti-Viral Immunity Laboratory, Department of Microbiology and Immunology, University of Melbourne. For intracellular analysis, cells were fixed and permeabilized with the Foxp3/Transcription Factor Staining Buffer Set (Thermo Fisher Scientific, Scoresby, VIC, Australia) and stained for FoxP3-BV421 (MF-14), IFNγ-Alexa Fluor 700 (XMG1.2) or TNFα-PE (TN3-19.12). Each staining cocktail also contained anti-CD16/32 (2.4G2) to minimize Fc receptor-mediated nonspecific antibody binding and the LIVE/DEAD Fixable Aqua Dead Cell Stain Kit (Invitrogen, Mulgrave, VIC, Australia) to discriminate non-viable cells. Samples were acquired using a BD LSRFortessa X-20 flow cytometer controlled by DIVA v9.0 software (BD Biosciences). Flow cytometric data were processed and quantified using FlowJo v10 software (Tree Star, Inc., Ashland, OR, USA) and presented relative to live or parent populations as indicated in the figures.

### 2.4. Quantification of Circulating Cytokines and C-Reactive Protein

Plasma cytokines and chemokines were quantified using the 8-Plex Mouse Luminex Discovery Assay (R&D Systems, Minneapolis, MN, USA; catalog number LXSAMSM-08). For each sample, 50 µL of plasma was analyzed in duplicate according to the manufacturer’s protocol. Measurements were acquired on a Bio-Plex 200 system (Bio-Rad, Gladesville, NSW, Australia), with 50 events counted per analyte region. Median fluorescence intensity was used for quantification.

C-reactive protein (CRP) concentrations in plasma and BALF were determined using the Mouse C-Reactive Protein/CRP DuoSet ELISA (R&D Systems). Duplicate 50 µL aliquots of each sample were added to pre-coated 96-well plates, and the assay was performed according to the manufacturer’s instructions. Absorbance was measured at 450 nm using a CLARIOstar microplate reader (BMG, Mornington, VIC, Australia). Sample concentrations were calculated from standard curves fitted using a four-parameter model.

### 2.5. Gene Expression Analysis by RT-qPCR

Total RNA was isolated from nasal and lung tissue homogenates using the RNeasy Mini Kit (Qiagen, Clayton, VIC, Australia). Between 0.5 and 1 µg of RNA was reverse-transcribed to cDNA with the High-Capacity cDNA Reverse Transcription Kit (Applied Biosystems, Foster City, CA, USA), following the manufacturer’s protocol. Target gene expression was assessed using pre-designed TaqMan assays (Life Technologies, Carlsbad, CA, USA) with TaqMan Fast Advanced Master Mix (Thermo Fisher Scientific). IAV RNA levels were assessed by amplification of the viral matrix (*M*) gene using custom-designed forward (5′-CTTCTTACTGAGGTCGAAACG-3′) and reverse (5′-CTGCAGTCCTCGTTCACTG-3′) oligonucleotides in conjunction with PowerUp SYBR Green PCR Master Mix (Thermo Fisher Scientific). Reactions were run on a QuantStudio 7 Flex Real-Time PCR System (Life Technologies) using an initial hold at 50 °C for 2 min and 95 °C for 2 min, followed by 40 cycles of 95 °C for 1 s and 60 °C for 20 s. Gene expression measurements were performed in triplicate. Relative expression was calculated using the comparative Ct method (2^−ΔΔCt^), normalized to the housekeeping gene *RPS18* and expressed relative to the corresponding uninfected controls.

### 2.6. Measurement of Antibody Levels

Antibody isotypes in BALF and plasma were quantified using the ProcartaPlex Mouse Antibody Isotyping Panel 7-Plex Kit (Thermo Fisher Scientific; catalog number EPX070-20815-901) as previously described [21]. The panel measured IgA, IgE, IgG1, IgG2a, IgG2b, IgG3 and IgM. For each measurement, 50 µL of undiluted BALF or appropriately diluted plasma was loaded in duplicate. Assays were conducted according to the manufacturer’s instructions, and median fluorescence intensity was determined using a Bio-Plex 200 instrument (Bio-Rad), with 50 counts acquired per region.

### 2.7. Statistical Analysis

Data are presented as mean ± standard error of the mean (SEM). Statistical analyses were performed in GraphPad Prism (version 10.1.2; GraphPad Software, Boston, MA, USA). Differences between experimental groups were assessed using two-way analysis of variance (ANOVA), followed by Tukey’s post hoc multiple-comparison testing.

## 3. Results

### 3.1. Innate Immune Cell Frequencies Differ Between WT and TLR7-Deficient Mice Across Respiratory and Systemic Tissues

**Figure 1:**
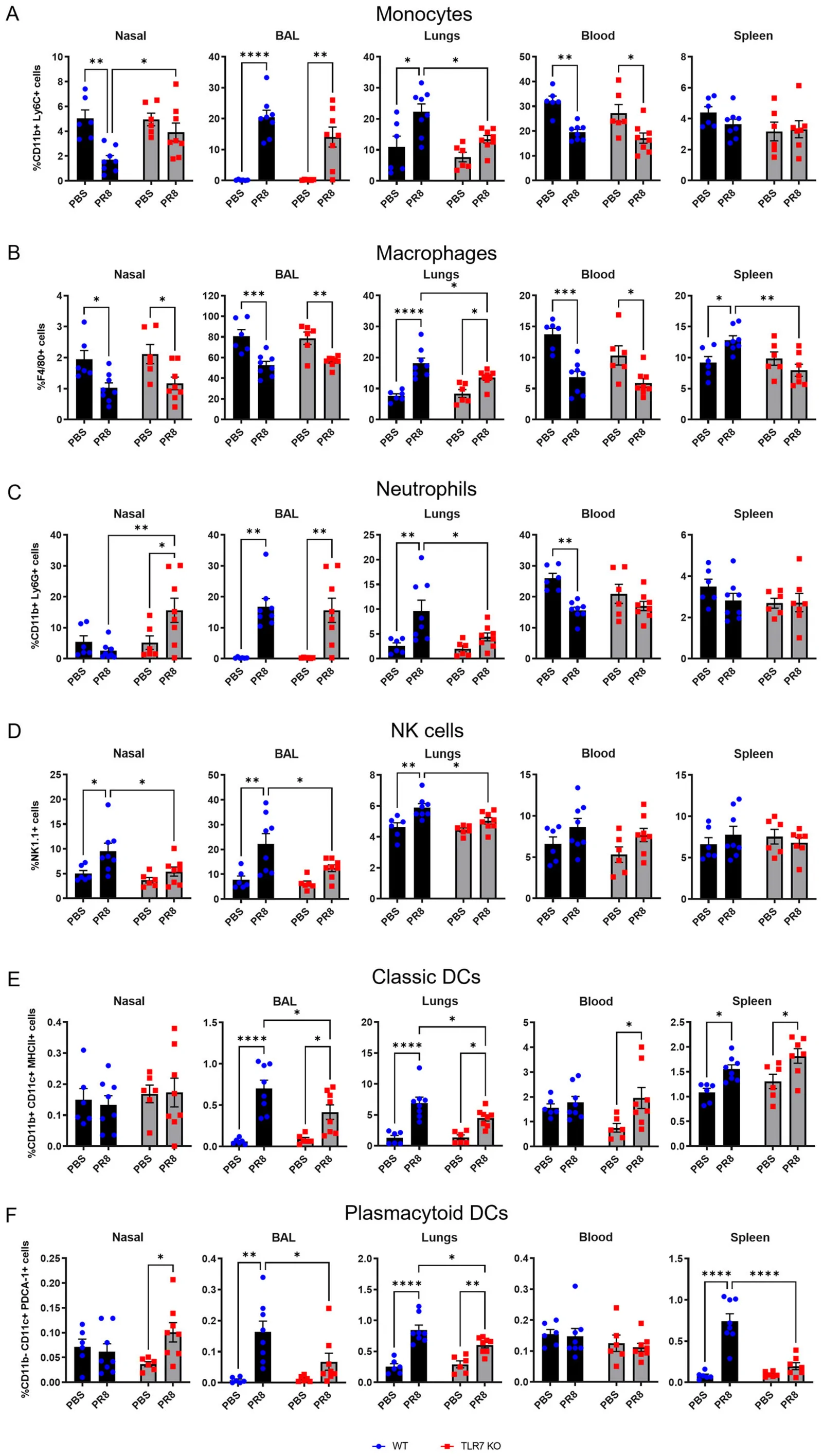
Differences in innate immune cell frequencies between WT and TLR7-deficient mice in respiratory and systemic compartments following IAV infection. WT C57BL/6 and TLR7 KO mice received intranasal PR8 virus (50 PFU) or PBS as an uninfected control. Respiratory and systemic tissues were collected at 7 days post-infection (dpi), and immune cell populations were quantified by flow cytometry: (**A**) Monocyte (CD11b+ Ly6C+): (**B**) macrophage (F4/80+), (**C**) neutrophil (CD11b+ Ly6G+), (**D**) NK cell (NK1.1+), (**E**) classic DCs (CD11b+ CD11c+ MHCII+) and (**F**) plasmacytoid DCs (CD11b- CD11c+ PDCA-1+). Frequencies are expressed as a proportion of live cells. Values represent mean ± SEM (*n* = 6–8 mice per group across two independent experiments). Differences were analyzed by two-way ANOVA with Tukey’s multiple-comparisons test (* *p* < 0.05, ** *p* < 0.01, *** *p* < 0.001, **** *p* < 0.0001).

We first characterized innate immune cell populations in nasal tissue, BAL, lung tissue, blood, and spleen following established IAV infection and compared with uninfected (PBS) control mice. In WT mice, infection altered the abundance of several innate immune populations across respiratory and systemic compartments. Ly6C+ monocytes decreased in the nasal tissue and circulation, while increasing in the BAL and lung (Figure 1A). F4/80+ macrophages decreased in the nasal tissue, BAL and blood but increased in the lung and spleen (Figure 1B). Ly6G+ neutrophils increased in the BAL and lung, while circulating neutrophils decreased (Figure 1C). The number of NK cells was higher in respiratory tissues but not in systemic compartments at this timepoint (Figure 1D). Both MHC-II+ classic DCs (Figure 1E) and PDCA-1+ pDCs (Figure 1F) increased in the BAL, lung and spleen with infection.

In TLR7 KO mice, infection similarly increased monocytes in the BAL but did not produce the increase observed in the lung. Macrophage accumulation in the lung and spleen was also absent or reduced compared with WT mice. Neutrophils increased in the BAL, but the lung increase observed in WT mice was not evident, while nasal neutrophils were increased in TLR7 KO mice. Unlike WT mice, infection did not induce significant increases in NK cells in these mice. Classic DCs increased following infection in the BAL, lung, blood and spleen of TLR7 KO mice, although responses in the respiratory compartments were significantly lower than those observed in WT mice.

A distinct genotype-dependent pattern was observed for pDCs across both respiratory and peripheral compartments. Infection increased pDC numbers in the BAL, lung and spleen of WT mice, whereas this response was significantly blunted in TLR7 KO mice. Conversely, pDC numbers were increased in the nasal tissue of infected TLR7 KO mice, where no corresponding increase was observed in WT mice. No significant infection- or genotype-dependent differences were detected in circulating pDCs.

Taken together, the compartmental analysis indicates that TLR7 deficiency did not uniformly alter innate immune responses across tissues (summarized in Figure S1A). Instead, genotype-dependent differences were most apparent within the lung, particularly among macrophages, neutrophils and classic DCs, while NK cell responses were more evident across the respiratory compartments. The contrasting pDC patterns between the nasal tissue and lower respiratory and splenic compartments further highlight the tissue-specific organization of the response. These findings indicate that the effect of TLR7 deficiency varied according to both immune cell population and anatomical compartment, rather than producing a generalized reduction in overall innate immune responses.

### 3.2. Differences in Compartment-Specific Adaptive Immunity and Systemic Cytotoxic T Cell Expansion in the Absence of TLR7

**Figure 2:**
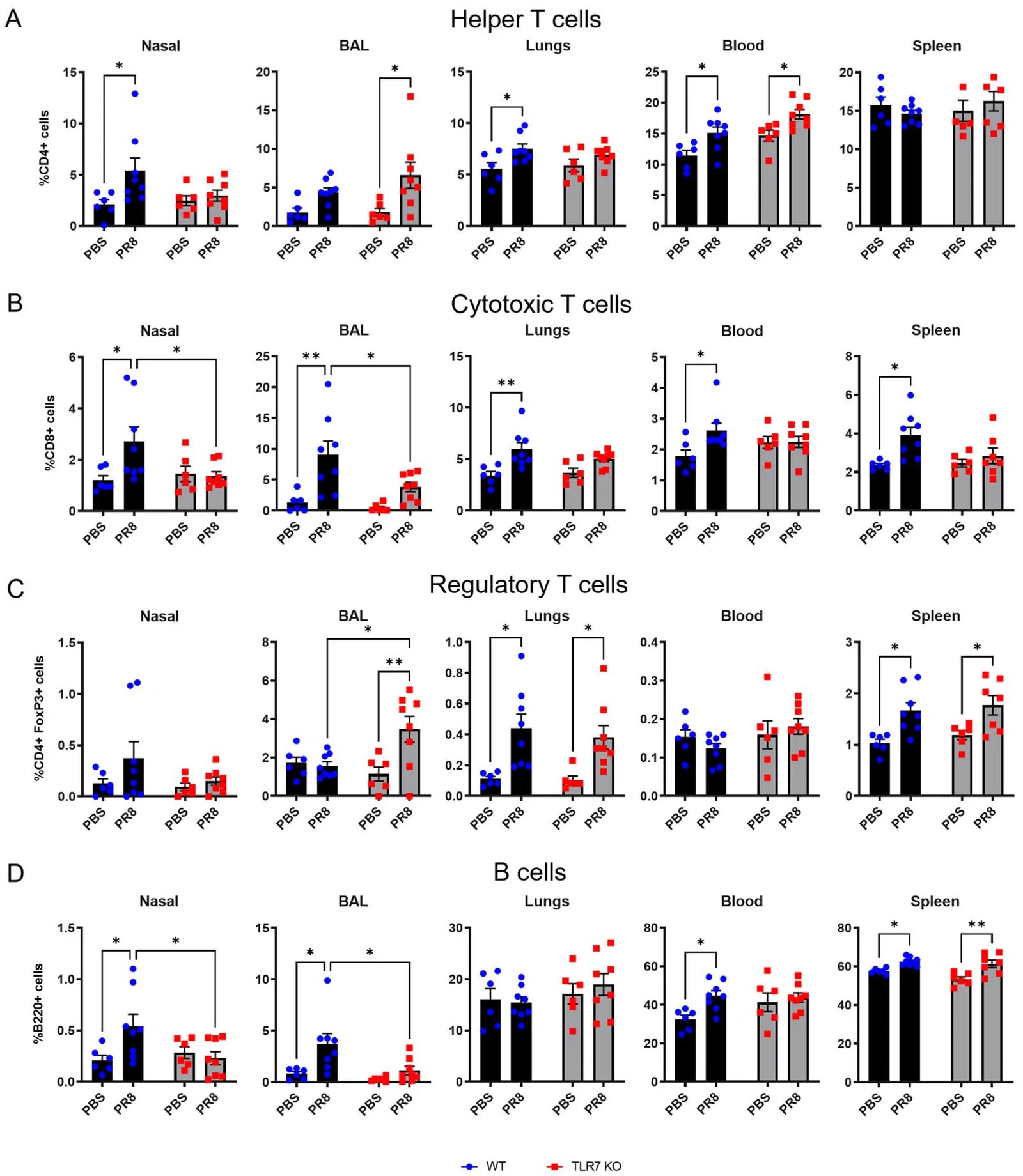
Differences in adaptive immune cell frequencies between WT and TLR7-deficient mice in the respiratory and systemic compartments following IAV infection. WT C57BL/6 and TLR7 KO mice were administered intranasal PR8 virus (50 PFU) or PBS (uninfected control), with tissues collected after 7 days. (**A**) Helper T cell (CD4+), (**B**) cytotoxic T cell (CD8+), (**C**) regulatory T cell (CD4+ FoxP3+) and (**D**) B cell (B220+) populations were measured by flow cytometry. Frequencies are reported relative to live cells. Values represent mean ± SEM (*n* = 6–8 mice per group across two independent experiments). Differences were analyzed by two-way ANOVA with Tukey’s multiple-comparisons test (* *p* < 0.05, ** *p* < 0.01).

We next examined adaptive immune populations across the respiratory and systemic compartments. In WT mice, IAV infection significantly increased CD4+ T cells in the nasal tissue and lung (Figure 2A), and CD8+ T cells across all tissues analyzed (Figure 2B). Treg numbers increased in the lung and spleen (Figure 2C), while B cells increased in the nasal tissue, BAL, blood and spleen (Figure 2D). These findings demonstrate that PR8 infection at 7 days post infection (dpi) induced adaptive immune responses across both respiratory and systemic compartments in WT mice.

In TLR7 KO mice, infection also increased several adaptive immune populations, although the magnitude and distribution of these responses differed from WT mice. CD4+ T cells increased in the blood but not in the nasal tissue or lung. Infection did not induce significant increases in CD8+ T cells residing in the respiratory and systemic tissues of TLR7 KO mice analyzed. Treg numbers increased in the lung and spleen, whereas B cells increased only in the spleen.

Comparison of infected WT and TLR7 KO mice identified pronounced genotype-dependent differences in the CD8+ T cell compartment. CD8+ T cell numbers were higher in WT mice than TLR7 KO mice across the nasal tissue, BAL, lung, blood and spleen. Similarly, B cell numbers were higher in WT nasal tissue, BAL and blood compared with TLR7 KO mice. The increased CD4+ T helper and Treg responses observed in the BAL of TLR7 KO mice was absent in WT mice. Overall, this compartmental analysis (summarized in Figure S1B) identifies that CD8+ T cell frequencies differed consistently between genotypes across respiratory and systemic tissues during PR8 infection, while other adaptive populations displayed more restricted patterns.

### 3.3. IAV Infection in TLR7-Proficient Mice Is Associated with Systemic Cytokinemia and T Cell Activation

We next investigated whether genotype-dependent differences in compartmental immune cell changes were associated with differences in systemic inflammatory cytokine responses during IAV infection. Consistent with our previous findings using an HK-x31 (H3N2) infection model [13], IAV PR8 infection at 7 dpi induced expression of *IFNB*, *IFNG*, and *TNFA* in nasal and lung tissues of WT mice (Figure S2). Significantly fewer viral transcripts were detected in the lungs of TLR7 KO mice, while viral transcript levels remained similar in nasal tissue.

**Figure 3:**
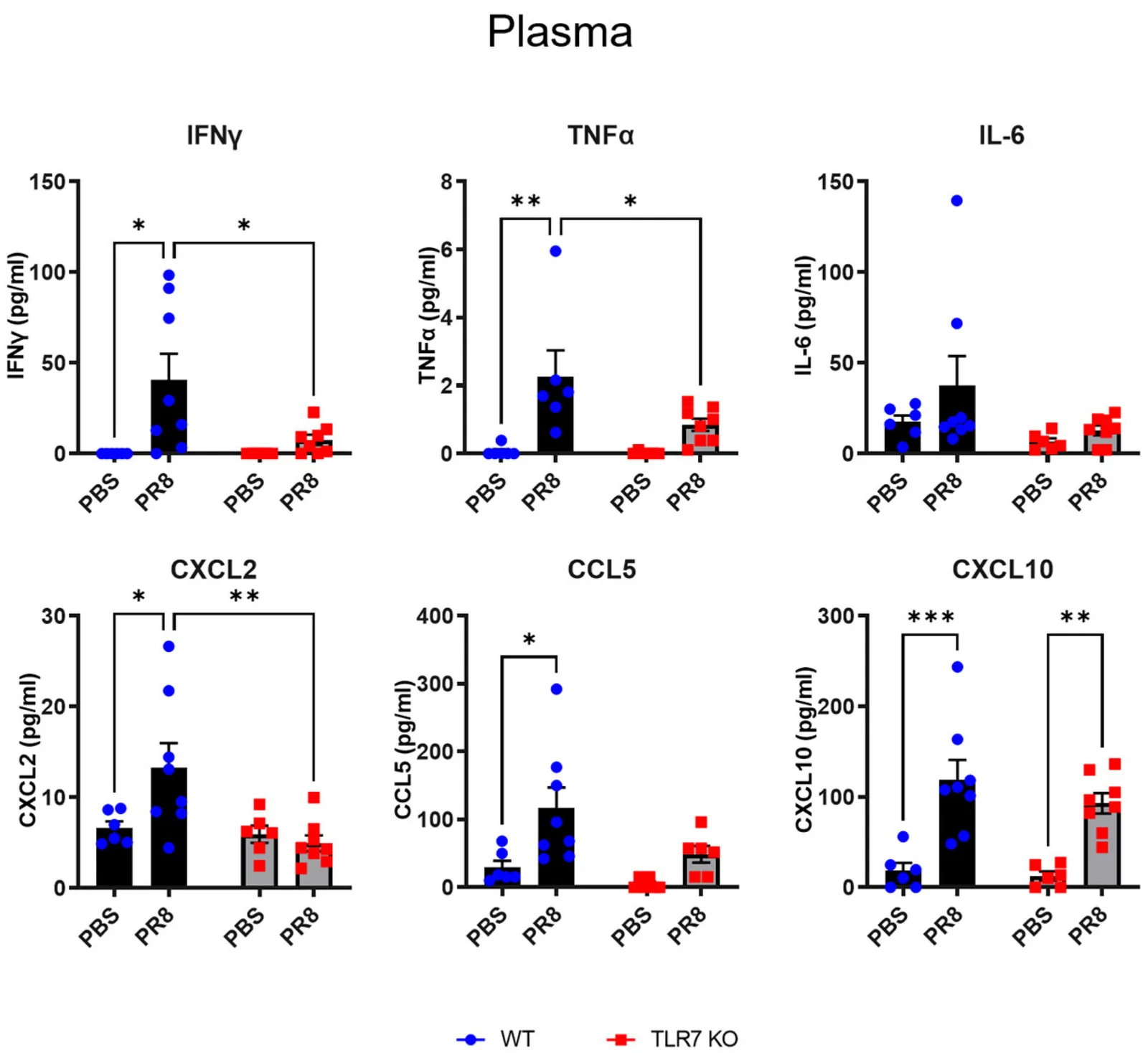
TLR7-deficient mice exhibit reduced circulating inflammatory cytokine and chemokine levels following IAV infection. WT C57BL/6 and TLR7 KO mice were administered intranasal PR8 virus (50 PFU) or PBS (uninfected control), and plasma was collected 7 dpi for multiplex quantification of inflammatory cytokines and chemokines. Values represent mean ± SEM (*n* = 6–8 mice per group across two independent experiments). Differences were analyzed by two-way ANOVA with Tukey’s multiple-comparisons test (* *p* < 0.05, ** *p* < 0.01, *** *p* < 0.001).

To assess systemic inflammation, we measured circulating cytokines and chemokines (Figure 3). Infection resulted in significant increases in plasma IFNγ, TNFα, CXCL2, CCL2, and CXCL10 in WT mice. These responses were significantly blunted in TLR7 KO mice, with the exception of CXCL10, which remained comparable between genotypes. These data indicate that the presence of TLR7 is associated with a broad systemic inflammatory cytokine response during IAV infection, in addition to localized inflammatory cytokine production in the BALF [18].

**Figure 4:**
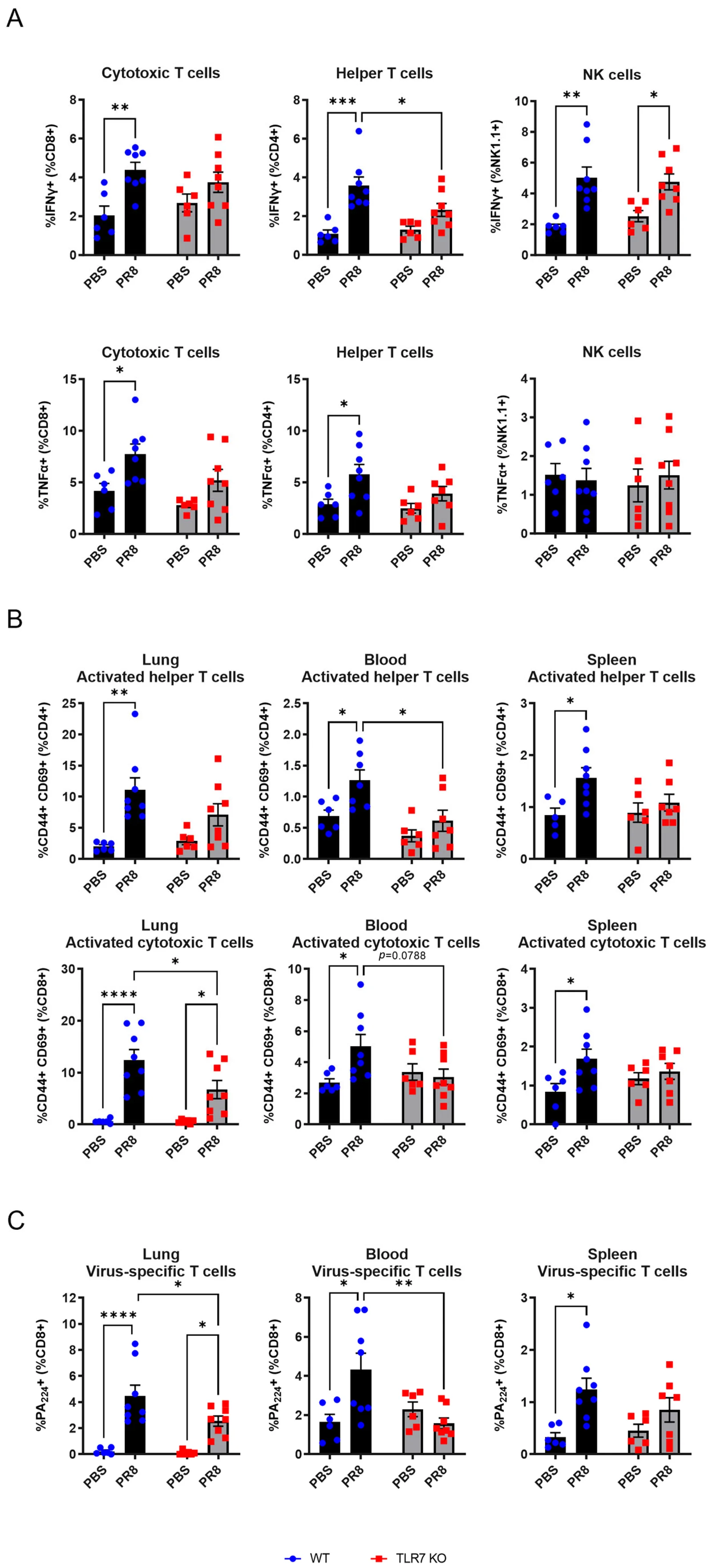
Markers of systemic T cell activation following IAV infection are elevated in TLR7-proficient mice. WT C57BL/6 and TLR7 KO mice were administered intranasal PR8 virus (50 PFU) or PBS (uninfected control), and tissues were collected after 7 days. (**A**) Intracellular IFNγ or TNFα levels from lung T cell subsets and NK cells are presented. (**B**) Antigen-experienced, activated T cells (CD44+ CD69+) or (**C**) virus-specific cytotoxic T cells expressing the epitope PA_224–233_ from lungs, blood and spleen are presented. Values represent mean ± SEM (*n* = 6–8 mice per group across two independent experiments). Differences were analyzed by two-way ANOVA with Tukey’s multiple-comparisons test (* *p* < 0.05, ** *p* < 0.01, *** *p* < 0.001, **** *p* < 0.0001).

Given the prominent increase in circulating IFNγ together with systemic CD8+ T cell expansion that was absent in TLR7 KO mice, we next examined whether these cytokine responses were linked to enhanced T cell activation. Intracellular cytokine analysis revealed increased IFNγ and TNFα production by both CD4+ and CD8+ T cells from infected WT mice, which was significantly reduced in TLR7 KO mice (Figure 4A). While NK cells also produced IFNγ, this response was unaffected by TLR7 deletion at this timepoint. Further analysis of activation markers demonstrated increased frequencies of CD44+CD69+ T cells in the lungs, blood, and spleen of WT mice, with significantly lower levels in TLR7 KO mice (Figure 4B). Consistent with this, virus-specific (PA224+) CD8+ T cells were more abundant in WT mice across both respiratory and systemic compartments, further supporting genotype-associated differences in antigen-specific cytotoxic T cell responses during infection.

Together, these findings demonstrate genotype-associated differences in systemic IFNγ and TNFα responses that coincide with altered antigen-experienced and virus-specific T cell responses during established IAV infection. This is consistent with the presence of TLR7 contributing to the magnitude of cytotoxic T cell response across both respiratory and systemic compartments.

### 3.4. Genotype-Associated Differences in CRP Levels Correlate with Clinical Disease Severity Following IAV Infection

**Figure 5:**
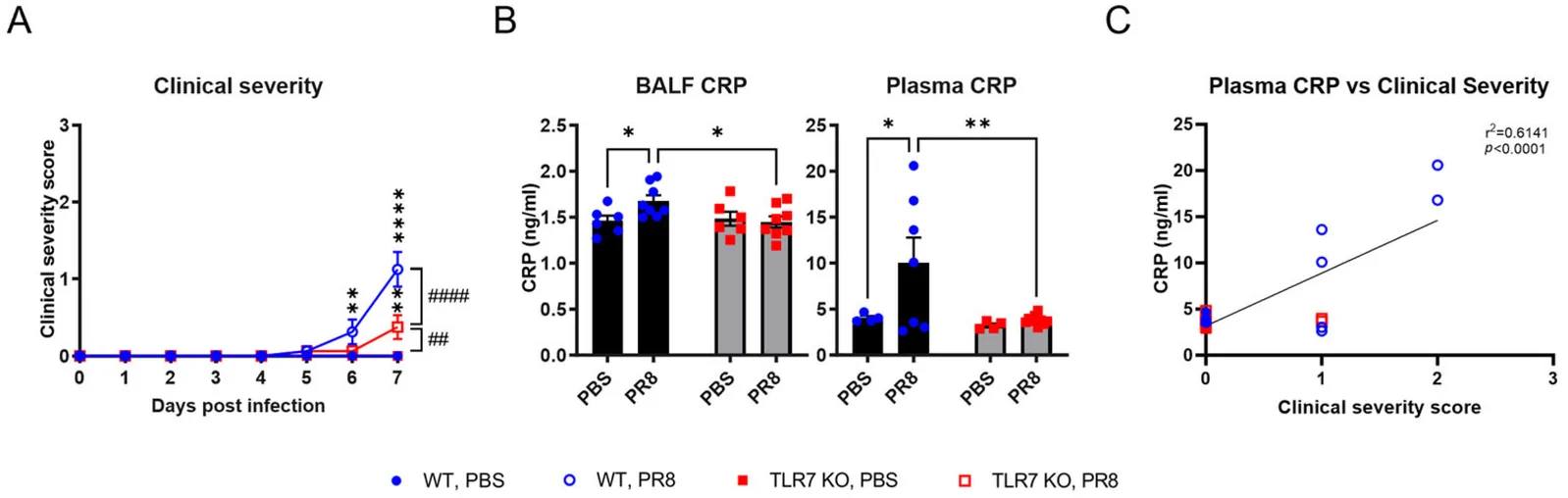
Differences in CRP concentrations between WT and TLR7 KO mice correlate with clinical disease severity following IAV infection. WT C57BL/6 and TLR7 KO mice were administered intranasal PR8 virus (50 PFU) or PBS (uninfected control), and analyses were performed 7 days post infection. (**A**) Clinical signs of disease, including body weight loss, lethargy, ruffled fur, and labored breathing, were assessed during the protocol and assigned a clinical severity score (0–3). (**B**) CRP levels were measured by ELISA from BALF and plasma. (**C**) Pearson correlation analysis showing the relationship between plasma CRP levels and clinical severity score in PR8-infected mice. Values represent mean ± SEM (*n* = 6–8 mice per group across two independent experiments). Statistical analysis was conducted using (**A**,**B**) two-way ANOVA followed by Tukey’s post hoc test for multiple comparisons (* *p* < 0.05, **/^##^ *p* < 0.01, ****/^####^ *p* < 0.0001) or (**C**) Pearson’s correlation coefficient. Asterisks (*) indicate comparisons between uninfected and infected mice within the same genotype, and hash (^#^) symbols indicate comparisons between infected WT and TLR7 KO mice.

PR8 infection in WT mice resulted in a greater clinical severity score compared with TLR7 KO mice (Figure 5A), consistent with previously reported differences in pulmonary histopathology in this infection model [22]. Given the observed differences in circulating cytokines and cytotoxic T cell responses between genotypes, we also examined CRP as a marker of systemic inflammation. CRP concentrations were elevated in the BALF and plasma of PR8-infected WT mice and were lower in infected TLR7 KO mice (Figure 5B). In addition, plasma CRP levels were positively associated with clinical severity among the animals analyzed (Figure 5C). These findings demonstrate that differences in CRP concentrations between experimental groups coincided with differences in clinical severity.

### 3.5. Differences in Mucosal and Systemic Antibody Isotype Profiles Between WT and TLR7 KO Mice

**Figure 6:**
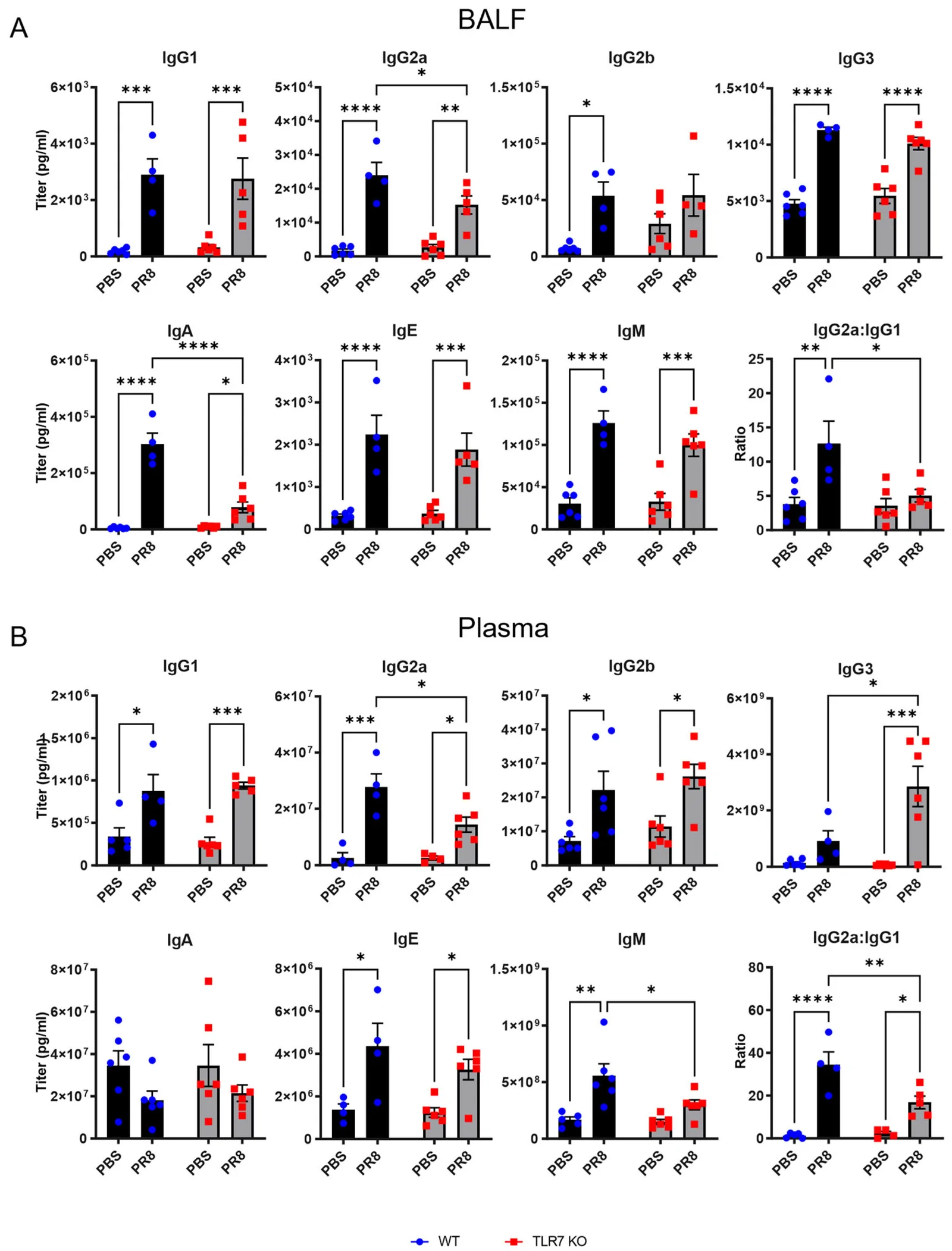
TLR7 deficiency is associated with differences in mucosal and systemic antibody levels following IAV infection. WT C57BL/6 and TLR7 KO mice were infected intranasally with PR8 virus (50 PFU) or administered PBS as uninfected controls. At 14 dpi, antibody isotypes in (**A**) bronchoalveolar lavage fluid (BALF) and (**B**) plasma were quantified using multiplex assays. Values represent mean ± SEM (*n* = 6–8 mice per group across two independent experiments). Differences were analyzed by two-way ANOVA with Tukey’s multiple-comparisons test (* *p* < 0.05, ** *p* < 0.01, *** *p* < 0.001, **** *p* < 0.0001).

Given the differences in B cell populations between genotypes, we next examined antibody isotype concentrations in the BALF and plasma following IAV infection. In BALF, PR8 infection was accompanied by increases in IgA, IgE, IgM and IgG subclasses in WT mice (Figure 6A). Antibodies were also detected in TLR7 KO mice, although IgA and IgG2a concentrations were significantly lower than those in infected WT mice. In the plasma, infection resulted in broad increases in multiple antibody isotypes across both genotypes (Figure 6B). However, plasma IgG2a and IgM concentrations were lower in TLR7 KO mice than in WT mice, whereas IgG3 concentrations were higher. The IgG2a-to-IgG1 ratio was also lower in TLR7 KO mice, indicating a difference in the relative abundance of these isotypes and suggesting a disrupted Th1-polarized humoral response. Collectively, these results demonstrate genotype-associated differences in antibody isotype concentrations in both mucosal and systemic compartments.

## 4. Discussion

In this study, we identified differences in innate and adaptive immune responses between WT and TLR7-deficient mice that extend beyond the respiratory tract to systemic immune responses during severe IAV infection. In addition to its established role in viral sensing, loss of TLR7 was associated with differences in multiple components of the antiviral response, including myeloid and antigen-presenting cell populations, cytotoxic T cell activation, inflammatory cytokinemia, acute-phase responses and humoral immunity. Importantly, these genotype differences were not uniform across respiratory and peripheral compartments, and differentially altered specific immune populations. Together with previously reported differences in disease severity, pulmonary histopathology and acute lung dysfunction [18,22], these findings are consistent with a role of TLR7-associated signaling in contributing to the broader systemic inflammation during severe IAV infection.

An important consideration in interpreting these genotype-associated immune differences is the potential contribution of altered viral control. Intranasal delivery of PR8 virus typically results in peak pulmonary viral burden between approximately 3 and 6 dpi [23], whereas the principal immunophenotyping in the present study was performed at 7 dpi. This timepoint represents an established stage of infection at which pulmonary inflammation, systemic cytokine responses and adaptive immune responses can be examined concurrently across multiple compartments. Here, viral transcript levels were reduced in the lungs of TLR7-deficient mice but remained comparable between genotypes in the nasal tissue. Accordingly, the lower systemic cytokine and adaptive immune responses observed in TLR7-deficient mice may therefore partly reflect differences in pulmonary viral or antigenic burden and the associated inflammatory stimulus. However, the present study cannot determine the extent to which altered viral burden accounts for the observed immune differences. Other potential contributors include differences in baseline physiology, development or behavior associated with the global KO model, as well as compensatory or redundant viral sensing through other pattern-recognition receptors, including TLR3 and RIG-I [24,25,26,27,28]. Because the study was not designed to distinguish these possibilities, the relative contributions of direct and indirect consequences of TLR7 deficiency remain unresolved. Further studies will be required to establish the exact mechanisms underlying the observed differences.

The selective impairment of lung macrophage and DC responses, splenic pDC/macrophage responses, virus-specific CD8+ T cell expansion and systemic cytokinemia is consistent with a broader role for TLR7 and the compartmental organization of immune responses during IAV infection. The contrasting pDC patterns between the nasal compartment and the lower respiratory and splenic compartments further emphasize the tissue-specific nature of these responses. Specifically, the increased pDC frequencies in the nasal compartment but reduced pDC accumulation in the BALF, lung and spleen of TLR7 KO mice suggest an altered distribution of pDCs across respiratory and peripheral compartments. Since pDCs promote type I IFN production and T cell priming [29], this reduced pDC abundance in the lung and spleen of TLR7-deficient mice is likely to result in the observed attenuated adaptive responses in these animals, namely diminished activation of antigen-experienced and virus-specific CD8+ T cells, as well as reduced intracellular IFNγ production. Such findings support an important role for TLR7-associated signaling in the broader respiratory–systemic immune response during established IAV infection, although longitudinal studies capturing earlier stages of infection will be required to define the temporal evolution of these responses.

Our data further identify a systemic IFNγ response that was associated with the genotype-dependent differences in cytotoxic T cell activation. Among the lymphocyte populations examined in this model, CD8+ T cell responses showed the most consistent differences between infected WT and TLR7-deficient mice across respiratory and systemic compartments, together with reduced intracellular IFNγ production by T cells. These findings implicate TLR7-associated signaling in the development of systemic CD8+ T cell responses during established IAV infection. While NK cells also produced IFNγ, their IFNγ response did not differ significantly between genotypes at the timepoint examined. Previous work at earlier stages of IAV infection (3 to 4 dpi) showed delayed NK activation and IFNγ in TLR7 KO mice [10]. We also observed reduced NK cell accumulation in respiratory tissues of TLR7 KO mice at day 7, indicating that genotype-associated differences in NK cell responses may persist beyond the earlier phase of infection. These findings are consistent with the established role of TLR7 in promoting Th1-polarized immunity and suggest that TLR7-associated differences extend to systemic inflammatory effector responses during established infection.

Importantly, this cytotoxic response has both protective and pathogenic implications. Effective CD8+ T cell immunity contributes to viral control, yet excessive cytotoxic T cell activation during respiratory viral infection can also contribute to tissue injury and immunopathology [30,31,32]. In the present study, the greater T cell activation observed in WT mice occurred alongside higher circulating IFNγ, TNFα, CXCL2 and CCL2, together with elevated CRP in both BALF and plasma. These infection-induced changes in systemic responses were significantly suppressed in TLR7 KO mice. While NK cells also produced IFNγ, this response was not affected by TLR7 deficiency at the timepoint examined. Thus, the observed CD8+ T cell phenotype provides one potential cellular correlate of the systemic inflammatory response. Overall, these findings support a relationship between the magnitude of cytotoxic and inflammatory responses observed in TLR7-proficient mice and the systemic inflammatory phenotype.

The positive correlation between systemic CRP levels and clinical severity provides further evidence that the systemic immune phenotype observed at day 7 was associated with severity of disease. CRP is a clinically recognized marker of systemic inflammation and tissue injury [33], and its elevation in WT mice occurred alongside increased systemic cytokines and activated CD8+ T cells. This is consistent with evidence that severe influenza is associated with systemic inflammation, vascular dysfunction and multi-organ involvement [34]. Supporting this broader systemic role, we recently demonstrated that TLR7 expression was associated with IAV-induced cardiovascular dysfunction during pregnancy [35], while elevated TLR7 expression has also been associated with increased CRP levels in inflammatory autoimmune disease [36]. Together, these observations suggest that TLR7-associated inflammatory responses may contribute to systemic disease manifestations in contexts where inflammatory regulation or vascular function is altered.

Furthermore, TLR7 deficiency was associated with altered mucosal and systemic antibody levels, consistent with a role for TLR7 expression in shaping antibody quality during IAV infection, both locally and systemically. Loss of TLR7 led to reduced BALF IgA responses, indicating a defect in mucosal antibody production at the site where secretory immunity is most relevant for respiratory viral defense. In the plasma, TLR7 deficiency reduced IgM, suggesting impaired early systemic humoral activation, and reduced IgG2a and the IgG2a-to-IgG1 ratio, consistent with impaired Th1-associated class switching. The elevated IgG3 response in TLR7 KO mice indicates that loss of TLR7 does not simply reduce antibody production, but instead alters the composition of the humoral response. While these observed differences cannot establish whether the altered isotype profiles reflect direct changes in B cell development, activation, antibody production or other indirect consequences of TLR7 deficiency, our findings support previous work implicating TLR7 in B cell maturation, antibody class switching and Th1-biased humoral immunity [11,12,37]. Such changes in antibody isotype composition can influence the quality and functional characteristics of antiviral humoral immunity.

Our findings may also have relevance beyond IAV infection, as TLR7 recognizes RNA from other ssRNA respiratory viruses and can shape the quality of antiviral immune responses [38]. For example, early TLR7-dependent type I IFN responses are protective in experimental SARS-CoV-2 infection [39], while impaired TLR7 signaling and defective type I IFN responses have been linked to severe COVID-19 [40,41]. Together with our findings, these observations support a broader role for TLR7 in antiviral immunity to ssRNA respiratory viruses. Importantly, the effects of TLR7 appear to depend on the timing and magnitude of activation and the specific viral and tissue context. For example, stimulating TLR7 prior to infection can prime an antiviral state and limit IAV-induced morbidity [14,19], whereas pharmacological suppression with IRS661 after established infection reduced cytokine production and pulmonary immunopathology in mice [17]. These findings provide a context for considering TLR7 as a potential therapeutic target to minimize systemic inflammatory disease during established infection but also highlight the need to distinguish early antiviral functions from later inflammatory effects. Our findings further raise the possibility of a trade-off associated with reduced TLR7 expression: although diminished systemic inflammation may limit acute inflammatory pathology, impaired dendritic cell and T cell responses could potentially affect the development of durable antiviral immunity [11]. Thus, any potential therapeutic modulation of TLR7 during infection will require careful evaluation of both the timing of intervention and its immediate and longer-term effects on antiviral immunity.

## 5. Conclusions

This study demonstrates that TLR7 deficiency is associated with significant alterations in the spatial distribution and magnitude of innate and adaptive immune responses during established IAV infection. These differences extend across the upper and lower respiratory tract, blood and spleen and include altered myeloid and DC responses, reduced systemic cytotoxic T cell activation, diminished circulating inflammatory cytokines, lower CRP levels and disrupted humoral immunity. The relatively small cohort sizes across the broad range of immune parameters examined limit statistical power and increase uncertainty around some of the observed differences and correlations; these findings should therefore be interpreted with appropriate caution. The compartment-specific nature of these findings highlights an association between TLR7 status and the broader respiratory–systemic immune response during severe IAV infection. However, as this study employed a global TLR7-deficient model, the observed differences cannot be attributed exclusively to direct effects of TLR7 signaling, and potential contributions from altered viral burden, compensatory immune-sensing pathways and other consequences of TLR7 deficiency remain to be established. Further investigation is therefore needed to clarify the mechanisms underlying these differences. Although therapeutic targeting was not evaluated in the present study, our findings provide a basis for future research examining whether and how modulation of TLR7 during infection could alter inflammatory responses while preserving effective antiviral immunity. Such investigations should assess multiple tissue compartments to capture the potential effects on both respiratory and systemic immune responses.

## Data Availability

The original contributions presented in the study are included in the article material; further inquiries can be directed to the corresponding author/s.

## Supplementary Materials

The following supporting information can be downloaded at https://www.mdpi.com/article/10.3390/cells15211922/s1. Figure S1: Immune cell analysis across respiratory and systemic compartments following IAV infection; Figure S2: TLR7 KO mice exhibit reduced proinflammatory gene expression in the nasal and lung tissues following IAV infection.

## Abbreviations

BALF, bronchoalveolar lavage fluid; CRP, C-reactive protein; dpi, days post-infection; FACS, fluorescence-activated cell sorting; IAV, influenza A virus; IFNG, interferon-γ; PBS, phosphate-buffered saline; PFU, plaque-forming unit; PR8, A/Puerto Rico/8/1934; ssRNA, single-stranded RNA; TLR7, Toll-like receptor 7; TLR7 KO, TLR7 knockout; TNF, tumour necrosis factor; WT, wild-type.
