# Supplementary material for: TLR7 Deficiency Reveals Compartment-Specific Respiratory–Systemic Immune Responses During Severe Influenza A Virus Infection
Source: Cells. 2026 Oct 22;15(21):1922. doi: 10.3390/cells15211922 (PMC13762330; doi:10.3390/cells15211922)
Supplement: Supplementary file 1 [file cells-15-01922-s001.zip › cells-4614169-supplementary.pdf]

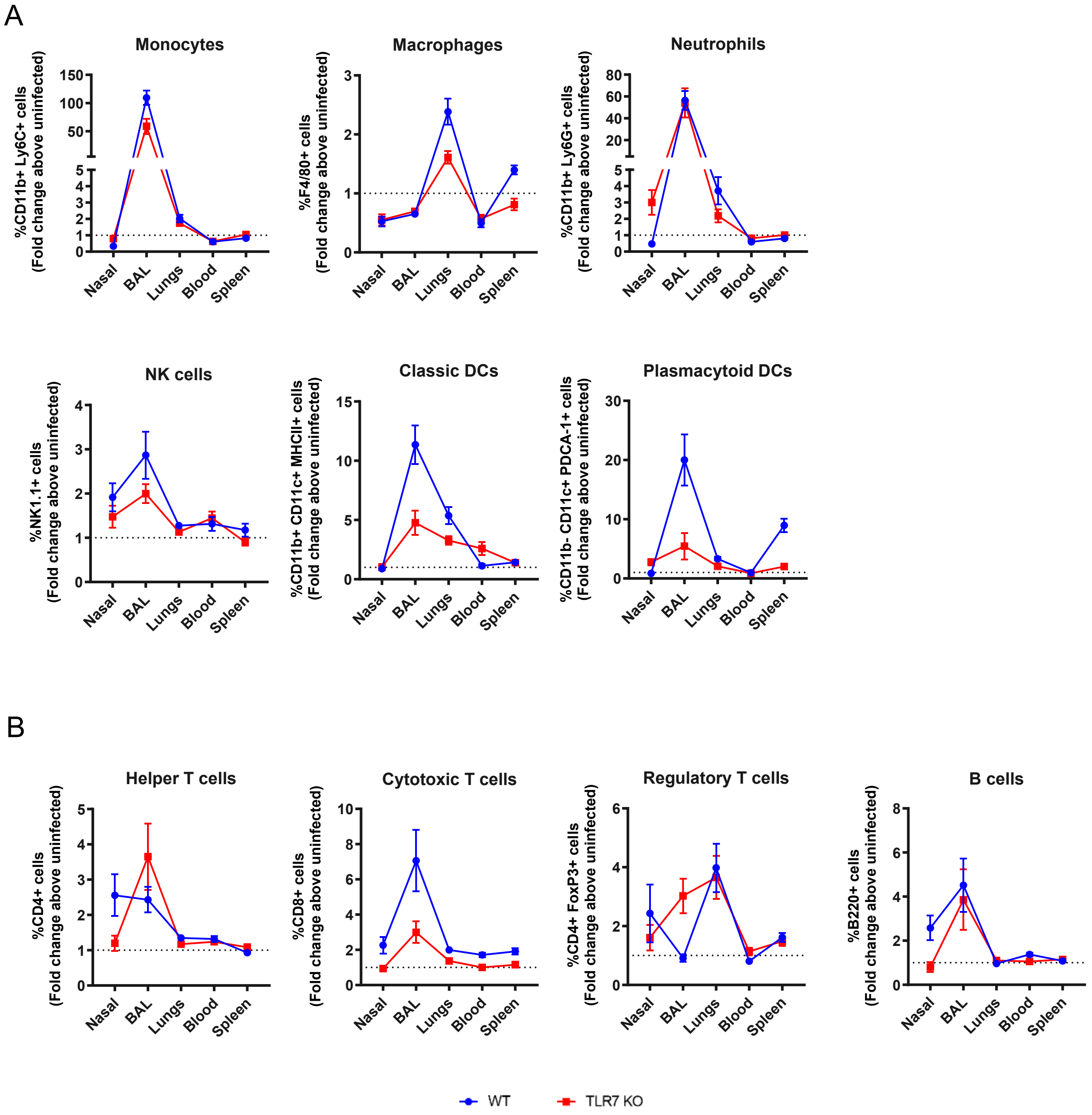

Figure S1. Immune cell analysis across respiratory and systemic compartments following IAV infection. WT C57Bl/6 or TLR7 KO mice were infected with PR8 (50 PFUs) or PBS (control), and respiratory and systemic tissues collected after 7 days. Fold changes of (**A**) innate and (**B**) adaptive immune cell type across tissues were calculated relative to the control group for each genotype. Data are expressed as mean ± SEM, n = 6-8 mice per experimental group from two independent experiments.

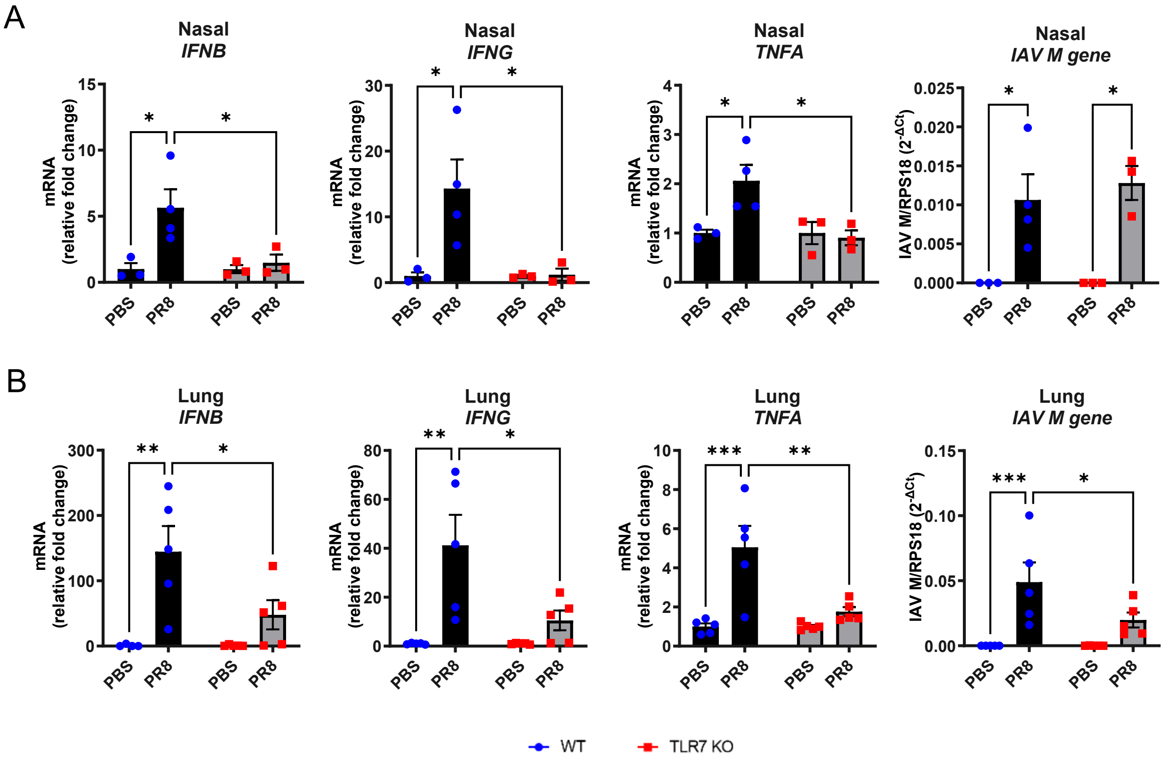

Figure S2. TLR7 KO mice exhibit reduced proinflammatory gene expression in the nasal and lung tissues following IAV infection. WT C57Bl/6 or TLR7 KO mice were infected with PR8 (50 PFUs) or PBS (control). Cytokine and viral gene expression analysis was performed in (**A**) nasal and (**B**) lung tissues by RT-qPCR after 7 days. Expression is presented relative to RPS18 housekeeping ($2^{-\Delta Ct}$) for viral burden or as a fold change above PBS controls for each genotype ($2^{-\Delta\Delta Ct}$) for cytokines. Data is expressed as mean ± SEM, n = 3-6 mice per experimental group. Statistical analysis was conducted using two-way ANOVA test followed by Tukey's post hoc test for multiple comparisons (*$p < 0.05$, **$p < 0.01$, ***$p < 0.001$).
